# Three cases of multicentric carpotarsal osteolysis syndrome: a case series

**DOI:** 10.1186/s12881-018-0682-x

**Published:** 2018-09-12

**Authors:** Peong Gang Park, Kee Hyuck Kim, Hye Sun Hyun, Chan Hee Lee, Jin-Su Park, Jeong Hae Kie, Young Hun Choi, Kyung Chul Moon, Hae Il Cheong

**Affiliations:** 1 Department of Pediatrics, Seoul National University Children’s Hospital, 101 Daehak-Ro, Jongno-Gu, Seoul, 03080 Korea; 2 Department of Pediatrics, National Health Insurance Service Ilsan Hospital, Goyang, Korea; 3 Division of Rheumatology, Department of Internal Medicine, National Health Insurance Service Ilsan Hospital, Goyang, Korea; 4 Department of Pathology, National Health Insurance Service Ilsan Hospital, Goyang, Korea; 5 Department of Radiology, Seoul National University College of Medicine, Seoul, Korea; 6 Department of Pathology, Seoul National University Hospital, Seoul, Korea; 7 Department of Pediatrics, Seoul National University College of Medicine, Seoul, Korea; 8 Research Coordination Center for Rare Diseases, Seoul National University Hospital, Seoul, Korea; 9 Kidney Research Institute, Medical Research Center, Seoul National University College of Medicine, Seoul, Korea

**Keywords:** Multicentric carpotarsal osteolysis syndrome, Idiopathic osteolysis, *MAFB* gene, Focal segmental glomerular sclerosis, Proteinuria

## Abstract

**Background:**

Multicentric carpotarsal osteolysis syndrome (MCTO) is characterized by progressive destruction and disappearance of the carpal and tarsal bones associated with nephropathy. MCTO is caused by loss-of-function mutations in the MAF bZIP transcription factor B (*MAFB*) gene.

**Case presentation:**

This report describes three unrelated patients with *MAFB* mutations, including two male and one female patient. Osteolytic lesions in the carpal and tarsal bones were detected at 2 years, 12 years, and 14 months of age, respectively. Associated proteinuria was noted at 4 years, 12 years, and 3 months of age, respectively. Kidney biopsy was performed in two of them and revealed focal segmental glomerulosclerosis (FSGS). One patient showed progression to end-stage renal disease, that is by 1 year after the detection of proteinuria. The second patient had persistent proteinuria but maintained normal renal function. In the third patient, who did not undergo a kidney biopsy, the proteinuria disappeared spontaneously. The bony lesions worsened progressively in all three patients. Mutational study of *MAFB* revealed three different mutations, two novel mutations [c.183C > A (p.Ser61Arg) and c.211C > G (p.Pro71Ala)] and one known mutation [c.212C > T (p.Pro71Leu)].

**Conclusion:**

We report three cases with MCTO and two novel *MAFB* mutations. The renal phenotypes were different among the three patients, whereas progressive worsening of the bony lesions was common in all patients. We also confirmed FSGS to be an early renal pathologic finding in two cases. A diagnosis of MCTO should be considered in patients with progressive bone loss concentrated primarily in the carpal and tarsal bones and kidney involvement, such as proteinuria.

## Background

Idiopathic osteolysis, or vanishing bone disease, represents a group of rare diseases characterized by destruction and resorption of affected bones with subsequent skeletal deformities and functional impairment. Hardegger et al. [1] classified this disorder according to the phenotypes and genotypes as follows: 1) type 1, hereditary multicentric osteolysis with dominant transmission; 2) type 2, hereditary multicentric osteolysis with recessive transmission; 3) type 3, nonhereditary multicentric osteolysis with nephropathy; 4) type 4, Gorham-Stout syndrome; and 5) type 5, Winchester syndrome, defined as a monocentric disease with autosomal recessive inheritance. Type 1, multicentric carpotarsal osteolysis syndrome (MCTO, type 1, Online Mendelian Inheritance in Man #166300), is clinically characterized by early childhood onset of progressive destruction and subsequent disappearance of the carpal and tarsal bones along with other large joints, such as the elbow and knee joints. Association of glomerulopathy is frequent, and many patients show progression to end-stage kidney disease.

After the causative gene for MCTO, that is, MAF bZIP transcription factor B (*MAFB*), was first identified by Zankl et al. [2], there have been several subsequent case reports with genetic diagnosis [3–6]. The *MAFB* gene encodes the v-maf avian musculoaponeurotic fibrosarcoma oncogene ortholog B (MafB), a basic leucine zipper transcription factor, which is known to be a regulator of various developmental processes, including osteoclastogenesis and renal development [7, 8].

We report three additional cases of MCTO with *MAFB* mutations, two of which had novel mutations.

## Case presentations

The clinical features of the three patients were summarized in Table 1.

**Table 1:** Summary of the clinical features of the three patients with multicentric carpotarsal osteolysis syndrome

Patients	Patient 1	Patient 2	Patient 3
Gender	Male	Female	Male
Current age	20 years	15 years	4 years
*MAFB* mutations	p.Pro71Ala	p.Ser61Arg	p.Pro71Leu
Bony lesions			
Onset age	2 years	12 years	3 months
Current state	Wheelchair-bound state	Progression of bony lesions	Wearing orthotics
Nephropathy			
Onset age	4 years	12 years	14 months
Mode of presentation	Proteinuria	Proteinuria	Proteinuria
Age at kidney biopsy	4 years	14 years	Not done
Pathological diagnosis	FSGS, NOS variant	FSGS, NOS variant	Not done
Current state	Kidney transplantation at the age of 5	eGFR 87.6 mL/min/1.73 m^2^	Normal renal function with spontaneous remission of proteinuria

*FSGS* focal segmental glomerulosclerosis, *NOS* not-otherwise specified, *eGFR* estimated glomerular filtration rate calculated using the Schwartz formula

### Patient 1

**Fig. 1:**
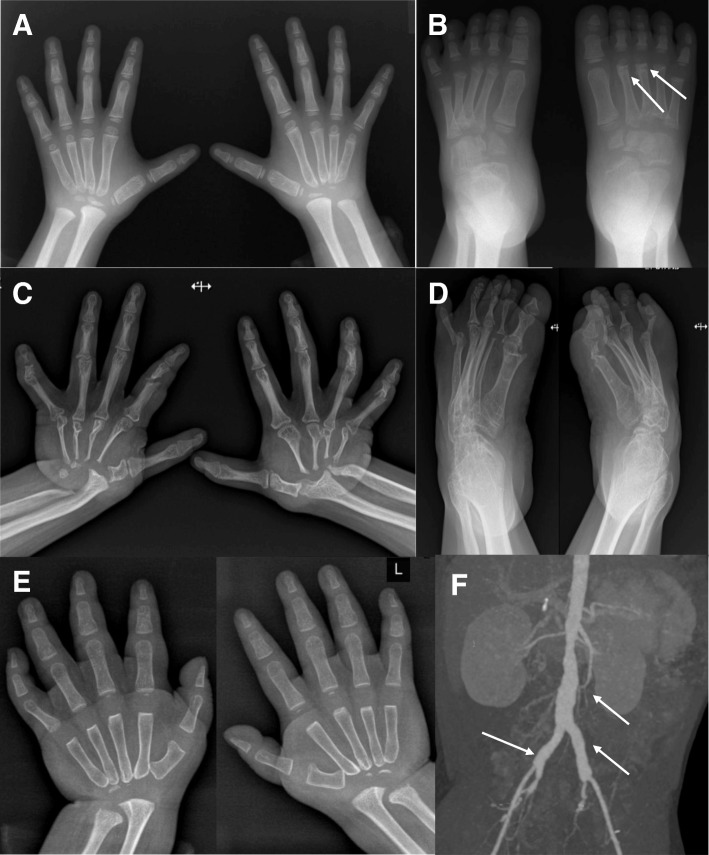
Radiological findings of 2 patients with multicentric carpotarsal osteolysis syndrome. In Patient 1, radiographs of the hands (**a**) and feet (**b**) obtained at 4 years old show severe bone resorption of the carpal and tarsal bones. Bone erosions are also noted in the proximal ends of metacarpal and metatarsal bones and the distal ends of the talus and calcaneus. Note that the distal metacarpal and metatarsal bones and the phalanges remained well-preserved at that time. Incidentally, there are fractures of the left 2nd and 3rd metatarsal necks (arrows). Follow-up radiographs of the hands (**c**) and feet (**d**) at the age of 20 show marked progression of bone resorption and associated joint contracture. In addition to the carpal and tarsal bones, the metacarpal and metatarsal bones, phalanges, distal radii, and ulnae are extensively involved. In Patient 3, radiographs of the hands (**e**) and feet (not shown) obtained at 1 year of age show multiple areas of osteolysis predominantly involving the carpal and tarsal bones. In addition, an abdominal aortic aneurysm was incidentally detected with renal ultrasonography (not shown), and a subsequently taken abdominal CT angiograph (**f**) confirmed fusiform aneurysms involving the infrarenal aorta and bilateral common iliac arteries (arrows)

**Fig. 2:**
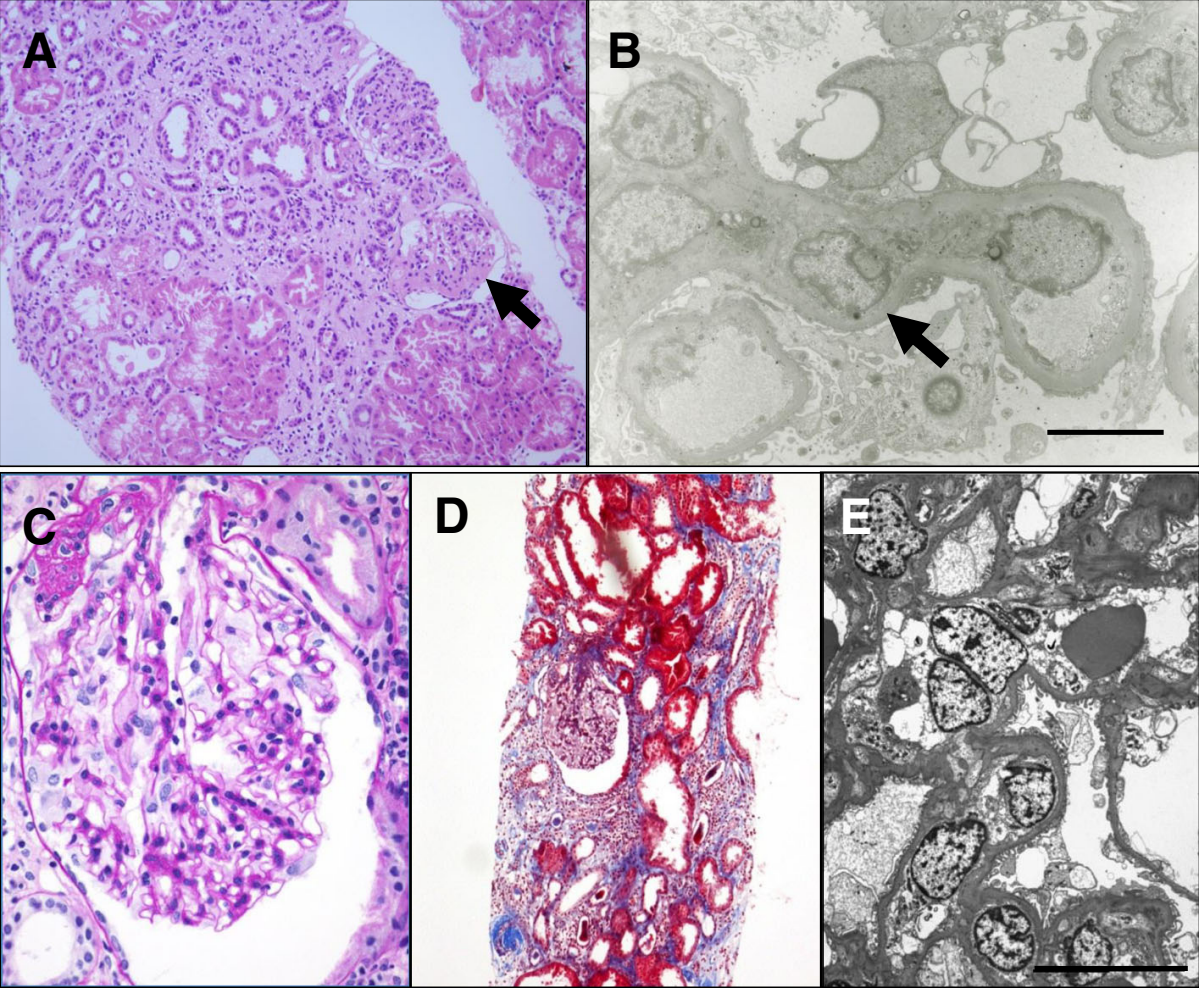
Renal pathological findings of 2 patients with multicentric carpotarsal osteolysis syndrome. In Patient 1, light microscopy (**a**) shows a glomerulus with segmental sclerosis (arrow), tubular atrophy, and interstitial fibrosis (hematoxylin and eosin, × 200). Electron microscopy (**b**) reveals wide effacement of foot process (arrow). (scale bar = 10 μm) In Patient 2, light microscopy shows a glomerulus perihilar segmental sclerosis (**c**, periodic acid–Schiff, × 400) and mild to moderate tubulointerstitial fibrosis with tubular atrophy (**d**, Masson’s trichrome, × 200), and electron microscopy (**e**) reveals wide effacement of the foot processes with occasional villous transformation and hydropic change of podocytes. (scale bar = 10 μm)

This male patient was born at term without any perinatal problems. At age 2 years, deformity of the left foot and pain in the right wrist developed. Radiologic studies revealed fragmentation of the left talonavicular joint and diffuse joint space obliteration in the right wrist joint (Fig. 1a and b). He was treated with oral prednisolone, methotrexate, and ibuprofen, with a clinical diagnosis of juvenile idiopathic arthritis (JIA), without any improvement. He also had a cleft palate and underwent corrective surgery at 3 years of age. At the age of 4 years, proteinuria was detected incidentally during a routine checkup. At that time, his blood pressure was normal. His parents did not have any skeletal lesions or nephropathy. Serum creatinine, albumin, and total cholesterol levels were 0.7 mg/dL, 3.1 g/dL, and 267 mg/dL, respectively. Serologic tests were all negative. A 24-h urine protein excretion was 2000 mg per day. He was treated with an oral steroid and enalapril, but the proteinuria persisted. A kidney biopsy performed 6 months later revealed focal segmental glomerulosclerosis (FSGS), not-otherwise specified (NOS) variant, with segmental and global sclerosis in 19 and 65% of the total glomeruli, respectively (Fig. 2a and b). His renal function deteriorated progressively, and chronic hemodialysis was started at the age of 5, 1 year after detection of the proteinuria. He underwent kidney transplantation 1 year after the initiation of hemodialysis. Meanwhile, multiple osteolytic lesions of the wrist, ankle, mandible and elbow joints worsened progressively. At 9 years of age, oral alendronate treatment (70 mg once a week) was started for his bony lesions. However, 5 months after starting the alendronate therapy, there was no evidence of improvement of the lesions or subjective symptoms. Currently, he is 20 years old, and his estimated glomerular function rate is 75.9 ml/min/1.73 m^2^. Severe joint narrowing with bilateral carpal and tarsal osteolysis persisted (Fig. 1c and d). He is wheelchair-bound because of the significant limitation of motion in his knee and ankle joints.

### Patient 2

A previously healthy female patient developed a deformity of the right thumb at the age of 12 years; radiological studies revealed bony erosion of the right scaphoid, trapezium, and triquetral bones and the distal end of the radius, with narrowing of the right wrist joint. She underwent correctional surgery. Although isolated 2+ proteinuria was detected on routine preoperative laboratory tests, no further study was done at that time. Other laboratory tests at that time revealed that her serum albumin and creatinine levels were 3.8 g/dL and 0.59 mg/dL, respectively, and a 24-h urine protein excretion was 1198 mg per day. At the age of 14, proteinuria was detected again on an annual school urinary screening and she was then evaluated at a hospital. Her blood pressure was normal. Serum creatinine, albumin, and total cholesterol levels were 0.73 mg/dL, 3.8 g/dL, and 180 mg/dL, respectively. The spot urine protein/creatinine ratio was 0.94 mg/mg. Serologic tests were all negative. A kidney biopsy revealed FSGS, NOS variant, with segmental and global sclerosis in 21 and 21% of the total glomeruli, respectively (Fig. 2c, d and e). She had swelling and tenderness in the right carpal and metacarpal joints as well as multiple proximal interphalangeal joints. Radiographs of the right hand revealed progression of the previous bone lesion with newly developed erosions at the 1st and 3rd metacarpal heads and the 1st proximal and distal phalangeal bones. The left hand was relatively unaffected. Laboratory tests, including erythrocyte sedimentation rate, C-reactive protein, rheumatoid factor, anti-citrullinated protein antibody, and HLA-B27, were all negative. She was treated with ibuprofen for a clinical diagnosis of JIA, but without any symptomatic improvement. She was referred to Seoul National University Children’s Hospital and was diagnosed with MCTO using genetic testing. At the last follow-up, she was 15 years old. The urine protein/creatinine ratio was 0.46 mg/mg while taking enalapril. The serum creatinine and albumin levels were 0.96 mg/dL (estimated glomerular filtration rate 87.6 mL/min/1.73 m^2^) and 4.6 g/dL, respectively.

### Patient 3

This male patient was born at term without any perinatal problems. At 3 months of age, he developed tenderness of his hands and feet without other focal inflammatory signs. At 6 months old, he developed contractures and limitation of motion of multiple joints including his wrist, ankle, elbow, and knee joints. At 14 months old, he could not stand even with assistance and he was referred to our hospital. Radiological studies revealed delayed secondary ossification with multiple osteolysis in the carpal and tarsal bones (Fig. 1e). Urinalysis revealed 1+ albuminuria and the spot urine protein/creatinine ratio was 0.84 mg/mg. Serum creatinine, albumin, and total cholesterol levels were 0.30 mg/dL, 4.5 g/dL, and 158 mg/dL, respectively. A kidney ultrasonogram revealed diffusely increased renal parenchymal echogenicity and an incidental finding of an abdominal aortic aneurysm. Subsequent abdominal CT angiography showed aneurysmal changes in the infrarenal aorta and bilateral common iliac arteries (Fig. 1f). All other laboratory tests indicating associated vasculitis were negative. However, the proteinuria resolved spontaneously 8 months after the initial detection and he did not undergo a renal biopsy. At the last follow-up at the age of 4 years, he had normal renal function without proteinuria. However, multiple osteolytic lesions progressed in the carpal and tarsal bones, and he needed to wear orthotics on his upper and lower limbs owing to severe joint contracture.

### Mutational analyses of the MAFB gene

Genomic DNAs of the patients and their available family members were isolated from their peripheral blood leukocytes. Entire coding regions of the *NAFB* gene were amplified via polymerase chain reaction and directly sequenced. All the primers for polymerase chain reaction were designed to start from intronic sequences, and the primer sequences are available upon request.

**Fig. 3:**
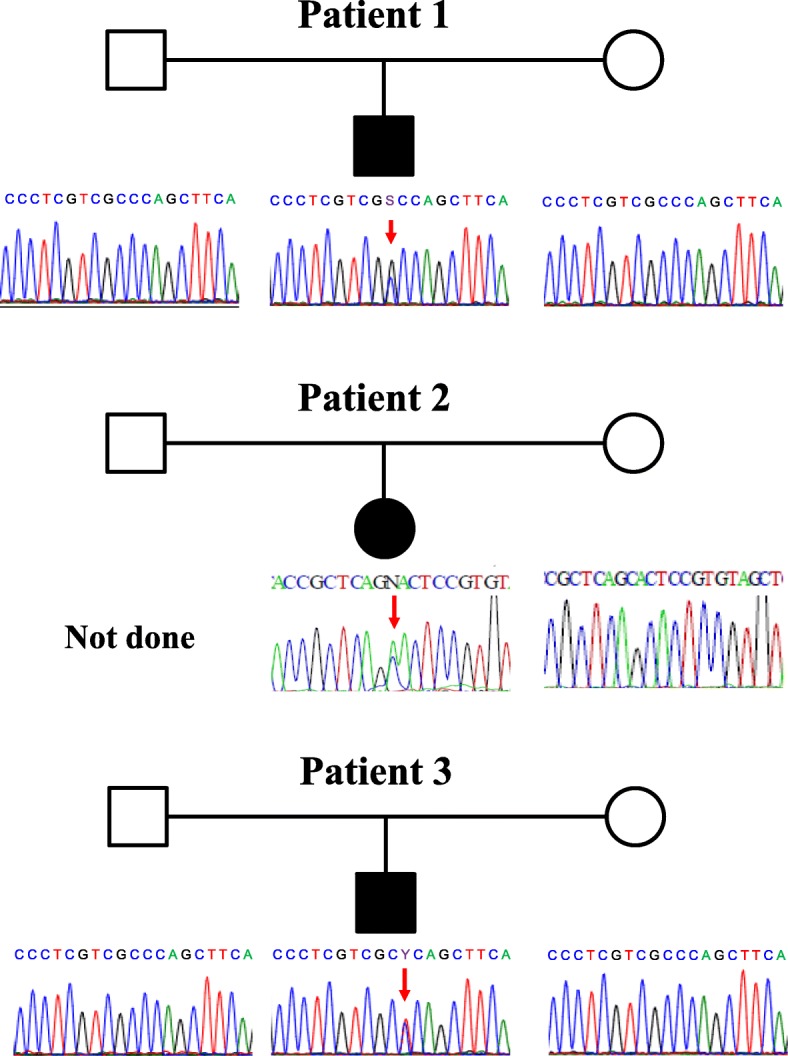
Pedigrees and sequence chromatograms of the patients and their parents. Patients 1 and 3 carry a de novo heterozygous mutation of c.211C > G (p.Pro71Ala) and c.212C > T (p.Pro71Leu) in the *MAFB* gene, respectively. Patient 2 carries a heterozygous *MAFB* mutation, c.183C > A (p.Ser61Arg). Her mother does not carry the mutation and her father’s sample was unavailable

A mutational study of the *MAFB* gene of Patient 1 and his parents revealed a de novo heterozygous mutation, c.211C > G (p.Pro71Ala). Patient 2 carried a heterozygous mutation, c.183C > A (p.Ser61Arg). Her mother did not carry the mutation and her father’s sample was unavailable. Patient 3 had a de novo heterozygous mutation, c.212C > T (p.Pro71Leu). (Fig. 3) Two of the mutations (p.Ser61Arg and p.Pro71Ala) were not listed in the Human Gene Mutation Database (HGMD® Professional 2017.4, https://portal.biobase-international.com/hgmd/pro/start.php), i.e., these were novel mutations. Neither mutation was found on the ExAC Browser (http://exac.broadinstitute.org/) nor was either mutation found on the 1000 Genomes Browser (http://www.internationalgenome.org/1000-genomes-browsers/) database. The MutationTaster application (http://mutationtaster.org) predicted these variants to be disease-causing. Functional studies of the novel mutations were not performed.

## Discussion and conclusions

We report three cases of MCTO with *MAFB* mutations. MCTO is a rare hereditary skeletal dysplasia characterized by progressive bone resorption, predominantly affecting the carpal and tarsal bones. Its onset is usually in early childhood and often progresses to severe bone destruction and multiple joint deformities. As it usually begins with joint pain and swelling, it is frequently misdiagnosed as polyarticular JIA [9]. However, it rarely responds to anti-rheumatic therapy and the subsequent clinical and radiographic appearances are characteristic [2]. Because the treatment of JIA includes non-steroidal anti-inflammatory drugs that should be avoided in patients at risk of nephropathy, a diagnosis of MCTO should be considered early in patients with carpal and tarsal osteolysis [4]. Two of our patients were initially misdiagnosed with JIA.

Kidney involvement is frequent, with an initial manifestation of proteinuria followed by progressive kidney failure. To date, a total of 31 probands with genetically confirmed MCTO have been reported [2–6]. Among them, 21 probands had renal involvement, including 13 with end-stage renal disease. Although renal biopsy obtained at a late stage of this disease reveals nonspecific findings of glomerulosclerosis and severe tubulointerstitial fibrosis, there have been only a few case reports with early renal biopsies that revealed findings of FSGS [6, 10, 11] In addition, a renal biopsy of a 2-year-old child without a genetic diagnosis revealed normal light microscopic findings, but abnormal fusion of the podocyte foot processes and discrete abnormalities of the glomerular basement membrane on electron microscopic examination [12]. Two of our patients had FSGS without glomerular basement membrane abnormalities on kidney biopsies, and one of them progressed to end-stage renal disease within 1 year after the detection of proteinuria. The remaining one patient showed spontaneous remission of the proteinuria.

Patients with MCTO may also have corneal opacity [5] or subtle craniofacial abnormalities, including triangular faces, micrognathia, maxillary hypoplasia, and consequent exophthalmos [13]. Our patients did not have any craniofacial or eye abnormalities, except for cleft palate in 1 of the patients. Although cleft lip and/or palate have not been reported as a phenotype of MCTO, significant association of cleft lip with and without cleft palate with multiple genetic variants near the *MAFB* gene was found by a recent genome-wide association study [14]. Patient 3 in our study also had aneurysmal changes in the aorta and common iliac arteries. However, there has been no report of MCTO complicated by an aortic aneurysm. Prior to identification of the causative gene, Bennett et al. [15] speculated that renal involvement and possibly osteolysis, results from a primary vascular disease since similar vascular changes of arteriolar thickening have been described in coronary vessels, skin, and the synovial cartilage. However, in our study, there was no significant vascular change in the early renal biopsy specimens of Patients 1 and 2. Therefore, the vascular changes, at least in the renal tissue, may not be a primary lesion but a late lesion. MafB attenuates macrophage apoptosis, which is associated with atherosclerotic plaque instability, and therefore, it may also be associated with the development of atherosclerosis and plaque vulnerability [16, 17]. However, none of our patients had hypertension or hypercholesterolemia, except for Patient 1 who had a high serum cholesterol level which may be due to nephrotic-range proteinuria.

We found three different *MAFB* mutations, including two novel mutations (p.Ser61Arg and p.Pro71Ala). To date, 15 different *MAFB* mutations causing MCTO have been listed in the Human Gene Mutation Database. All of the mutations, including two novel mutations detected in our study, were missense mutations and lie within a short amino-terminal region (from the 54th serine to the 71st proline residue) coding for a transactivation domain of the MafB protein. These findings suggested that MCTO is caused by only a few domain-specific mutations in *MAFB* [5]. Lack of nonsense or other truncating mutations suggested a dominant-negative pathogenesis [5]. Although functional studies of the novel mutations were not performed, there were several indirect pieces of evidence to support that these mutations might be pathogenic: 1) these mutations were not detected in normal subjects, 2) in silico tests predicted these mutations to be disease-causing, and 3) these mutations were located in a region where previously reported pathogenic mutations were clustered.

Since the causative gene, *MAFB*, was identified, the pathogenesis of MCTO began to be elucidated. Osteoclastogenesis is controlled by the receptor activator of nuclear factor κB ligand (RANKL)-signaling pathway, and MafB negatively regulates RANKL-induced osteoclastogenesis [7]. Thereafter, defective MafB would affect RANKL-mediated osteoclast differentiation, causing an imbalance in bone remodeling, apparently leading to the significant osteolysis [7]. However, it is not clear why the bony changes in MCTO are seen predominantly in the carpal and tarsal bones [2]. MafB is also known to play a critical role in normal development of podocyte foot processes and is expressed in both the podocyte late in renal development and in the mature glomerulus [8]. Homozygous *MafB*^−/−^ null mutant mice have renal dysgenesis, including loss of normal foot processes of podocytes, abnormal glomerular differentiation with decreased mature glomeruli, tubular dysgenesis due to abnormal tubular survival, and renal cysts [8].

Currently, there are no effective treatment options for the bone lesions and nephropathy of patients with MCTO patients. As inferred from molecular pathogenesis, blocking the RANK/RANKL pathway or disturbing osteoclast activity may be helpful. Bisphosphonates may be effective for treating or stopping the formation of the bony lesions by interrupting osteoclast activity. In a previous report [18], bisphosphonates seemed to slow down, but not stop, the progression of bone destruction in 2 patients. In our study, alendronate showed no definite improvement of the bony lesions in 1 patient. Although further study should collect more clinical data on the effect of bisphosphonates, bisphosphonate itself is nephrotoxic and should be used carefully. Denosumab, an anti-RANKL antibody, may be considered another possible drug, considering its mechanism. A single dose of denosumab (60 mg) was able to partially improve the magnetic resonance images 9 months later in a 13-year-old patient with a p.Ser54Leu mutation [6]. Regarding nephropathy, traditional oral steroids and/or other immunosuppressive drugs had no effect, although there was an exceptional case in which successful treatment with cyclosporine A was reported [11].

In conclusion, we report three cases with MCTO and two novel *MAFB* mutations. The renal phenotypes were different among these 3 patients, while progressive worsening of the bony lesions was common. We also confirmed FSGS to be an early renal pathologic finding in 2 cases. A diagnosis of MCTO should be considered in patients with progressive bone loss concentrated primarily in the carpal and tarsal bones and kidney involvement, such as proteinuria.

## Abbreviations

FSGS: Focal segmental glomerulosclerosis
JIA: Juvenile idiopathic arthritis
*MAFB*: MAF bZIP transcription factor B
MafB: v-maf avian musculoaponeurotic fibrosarcoma oncogene ortholog B
MCTO: Multicentric carpotarsal osteolysis syndrome
NOS: Not-otherwise specified
RANKL: Receptor activator of nuclear factor κB ligand
